# Care needs and self-induced measures of women with postpartum pelvic floor disorder- Results of a social media-based survey of 2930 women

**DOI:** 10.1007/s00404-024-07369-6

**Published:** 2024-02-14

**Authors:** Fabinshy Thangarajah, Johannes Soff, Caroline Lenz, Janice Jeschke, Jan Kössendrup, David Papior, Carsten Hagenbeck, Verena Kirn, Nadine Scholten

**Affiliations:** 1 Department of Gynecology and Obstetrics, Medical Faculty, Hufelandstr. 55, 45147 Essen, Germany; 2 Department of Gynecology and Obstetrics, Medical Faculty, Kerpener Str. 34, 50931 Cologne, Germany; 3 Faculty of Medicine and University Hospital Cologne, Institute of Medical Sociology, Health Services Research and Rehabilitation Science, Chair of Health Services Research, University of Cologne, Cologne, Germany; 4 Clinic for Gynecology and Obstetrics, Heinrich-Heine-University Düsseldorf, Moorenstr. 5, 40225 Düsseldorf, Germany; 5 Breast Center at the Department of Obstetrics and Gynecology, Heilig Geist Krankenhaus/Teaching Hospital of the University of Cologne, Cologne, Germany

**Keywords:** PFD, Care needs, Intervention needs, Information needs, Postpartum

## Abstract

**Introduction:**

Pelvic floor disorders (PFD) occur in about 40% of women after delivery. Less is known about the intervention and care needs of women with postpartum PFD. The aim of this analysis was to analyze care needs and self-initiated measures to strengthen the pelvic floor in postpartum women in relation to incontinence and sexual dysfunction. Furthermore, influencing factors for self-initiated measures were evaluated.

**Patients and methods:**

An anonymous online survey (via LimeSurvey) was conducted between September and October 2022 and distributed via social media (Instagram and Facebook). The survey explicitly addressed mothers with and without pelvic floor disorders up to 5 years postpartum (inclusion criteria). Validated instruments were employed to assess incontinence (ICIQ-SF) and sexual functioning (PISQ-IR: Condition Impact). The questions on the use of services and preventive measures, as well as on the interaction with a gynecologist, were based on self-developed items.

**Results:**

In total, 49.4% of the participants of the survey showed symptoms of urinary incontinence (UI). Furthermore, only 40.3% (*n* = 241) of women were actively asked by their gynecologists for the occurrence of UI or PFD among those who suffered from PFD. Overall, 79.3% of the participants of the survey with UI underwent measures to deal with the complaints. The ICIQ-SF Score was significantly associated with all self-induced measures. High School diplomas and academic degrees were associated with the use of love balls (*p* < 0.05).

**Conclusion:**

The results of the study show the unmet needs of postpartum women. PFD should be addressed more frequently in the outpatient setting. Furthermore, more systematic information about the treatment of PFD could help to address unmet information needs and improve interventions.

## What does this study add to the clinical work?

**Table:** 

Unmet needs of postpartum women with pelvic floor disorder are frequent and should be addressed in the outpatient setting.	

## Introduction

Postpartum pelvic floor disorder (PFD) defined as urinary, defecatory and sexual dysfunction occurs in about 40% of women after delivery [1]. Women with severe pelvic disorders can have access to specialized pelvic floor physical therapy. Therefore, a referral by the outpatient gynecologist is needed.

Besides the participation in postpartum courses, some women use electric or non-electric devices to treat PFD. Some of these devices are not financed by public health insurance. Current studies show that postpartum pelvic floor muscle training decreases the rate of urinary incontinence 6 months after confinement [2]. Furthermore, electrical devices seem to have a positive influence on the development of symptoms associated with PFD. For example, transvaginal electrical stimulation for as little as 5 sessions might be beneficial for helping postpartum women to control pelvic muscle contractions and to increase muscle strength [3]. Other studies report that physical therapy ± electrostimulation was the most effective non-surgical intervention [4].

However, there is limited information about PFD in the perinatal period [5]. Knowledge about postpartum PFD and therapeutic options among healthcare workers can vary [6]. Although knowledge among healthcare workers is available patients are not systematically asked about PFD-related symptoms [6]. Current data state that the awareness und knowledge about postpartum PFD among patients is limited and seems to be depended from the educational status [6]. Though the impression in occupational routine is that an amount of patients seem to be informed about PFD, but less is known about the intervention and care needs of women with postpartum PFD. Furthermore, there are less studies assessing self-induced measures of women with postpartum PFD.

The aim of this analysis was to analyze care needs and self-initiated compared to gynecologist-initiated measures to strengthen the pelvic floor in postpartum women in relation to incontinence and sexual dysfunction. Furthermore, influencing factors for self-initiated measures were evaluated.

## Population and methods

An anonymous online survey (via LimeSurvey) was conducted between September and October 2022 and distributed via social media (Instagram and Facebook). The survey explicitly addressed mothers with and without pelvic floor disorders up to 5 years postpartum (inclusion criteria). When possible, validated instruments were employed to assess incontinence (ICIQ-SF) and Sexual Functioning (PISQ-IR: Condition Impact). The questions on the use of therapeutic measures, as well as on the interaction with a gynecologist, were based on self-developed items. The questionnaire was developed in cooperation with clinicians, physical therapists and social scientists. A pre-test with the target group took place regarding comprehensibility and technical implementation. Statistical analysis was performed using STATA 15 and R. Patient characteristics were described using mean (SD) and range or count (percentages), as appropriate.

### Ethical votum

The survey received a positive ethics vote (no. 21-1542_1) from the Ethics Committee of the Medical Faculty of the University of Cologne. Financing was provided entirely from budgetary resources without external funding. Participation in the anonymous study was completely voluntary and possible after giving informed implicit consent. In free texts, it was pointed out not to provide any personal identifying information. After completion of the survey, it was possible to participate in a separate lottery for shopping vouchers.

### Questionnaire instruments

The scales and instruments used for these analyses are presented here.

#### ICIQ-SF international consultation on incontinence questionnaire-urinary incontinence short form

The ICIQ-SF is an internationally used instrument to assess incontinence and consists of 4 questions on the following dimensions: frequency or urinary incontinence, amount of leakage, overall impact of urinary incontinence, self-diagnostic item. The total scores ranged between 0 and 21. (The official German version of the instrument used can be found here: https://iciq.net/iciq-ui-sf).

#### PISQ-IR: pelvic organ prolapse/urinary incontinence sexual questionnaire, iuga-revised: condition impact

As a validated instrument, the PISQ-IR is suitable to evaluate women's sexual functioning with a focus on pelvic floor function [7] (https://www.iuga.org/resources/pisq-ir). Here, the dimension "Global Impact" was used, which addresses the limitation of sexuality based on pelvic floor disorders and differentiated between sexually active and sexually inactive women.

### Details of the last birth

The questions in relation to the last birth are self-developed and cover the mode of birth (Caesarean section, spontaneous vaginal, vacuum extraction and forceps), birth injuries (episiotomy and perineal laceration), birth weight and gestational age.

### Utilization of services and self-initiated measures

The use of services, as well as the self-implemented measures for the therapy of pelvic floor disorders were also surveyed by self-developed items. We posed the following questions: “Have you talked to your gynecologist about incontinence?” (yes/no), “Has your gynecologist actively addressed incontinence or descent problems?” (yes/no), “Would you like your gynecologist to actively address complaints such as incontinence or problems with prolapse?” (yes/no) and finally “With regard to my complaints, I have felt taken seriously by my gynecologist.” (not at all/little/somewhat/very). If the women had at least one point in the ICIQ-SF score, the following gynecologist-induced measures were questioned: Pelvic floor training, pessary therapy, physiotherapy, referral to a specialist. And the following self-induced measures were questioned: electric pelvic floor trainer, love/vaginal/kegel balls, books/internet resources for self-instruction of pelvic floor training. Self-induced measures were summarized for the multivariate models: 1. books and internet resources and 2. electric pelvic floor trainer and vaginal balls.

### Sociodemographic aspects

The current age of the respondents was surveyed, as well as their highest formal education attainment.

## Results

Overall, the data of 2.930 women were analyzed. The mean age was 32.9 years (18–49, SD 4.2) and on average 1.5 children were delivered (1–6, SD 0.7). Further characteristics can be found in Table 1. Participants were asked how often UI occurs. In total, 51.6% of the participants had no UI, whereas 49.4% had UI. Detailed answers concerning the ICIQ-SF are given in Table 2. The participants of the survey were asked how much urine leakage interferes with their everyday life. The answers were given on a scale between 0 (no deal) and 10 (great deal). The mean score was 2.9 (SD 2.0). The mean ICIQ-SF Score was 3 (SD 3.8). Fecal incontinence occurred in 162 out of 2819 (5.7%) women. We found that only 22.9% of the participants have ever talked to their gynecologist about PFD. Furthermore, only 40.3% (*n* = 241) of women were actively asked by their gynecologists for the occurrence of UI or PFD among those who suffered from PFD. However, 63.4% (*n* = 1193) would have liked to be asked by their gynecologist if UI or PFD symptoms occurred. 15.6% of those participants with UI or PFD did not feel taken seriously and 21.2% (*n* = 84) of the participants with UI or PFD felt taken less seriously by their gynecologist.

**Table 1:** Patient characteristics

Characteristic	N = 2,930^1^
Age	
Mean (SD)	32.9 (4.2)
Range	18.0, 49.0
Number of births (taking into account multiple births)	
Mean (SD)	1.47 (0.71)
Range	1.00, 6.00
Week of pregnancy (last birth)	
Mean (SD)	39.32 (3.61)
Range	1.00, 45.00
Level of education	
Certificate of secondary education	28 (1.1%)
General certificate of secondary education	238 (9.4%)
General/subject-related qualification for university entrance	678 (27%)
(Technical) university degree	1,581 (63%)
Injuries (last birth)	
Perineal tear	874 (30%)
Episiotomy	357 (12%)
Birth Procedures (last birth)	
Vaginal birth	1,904 (66%)
Cesarean section	716 (25%)
Labor induction	674 (24%)
Obstetrical forceps	25 (0.9%)
Vacuum extraction	354 (12%)
PISQ-IR—Condition Impact	
PISQ-IR Score: not sexually active—condition impact	1.49 (0.84)
PISQ-IR Score: sexually active—condition impact	3.62 (0.61)
Abnormal gynecological findings (during 2. examination after last delivery)	202 (7.7%)

^1^n (%), Mean (SD)

**Table 2:** ICIQ—SF: Scoring Questions

Characteristic	N = 2,930
How often do you leak urine?	
Never	1,478 (51,6)
About once a week or less often	932 (33%)
Two or three times a week	268 (9.4%)
About once a day	98 (3.4%)
Several times a day	78 (2.7%)
All the time	8 (0.3%)
How much urine do you usually leak?	
None	10 (0.7%)
A small amount	1,299 (94%)
A moderate amount	70 (5.1%)
A large amount	3 (0.2%)
Overall, how much does leaking urine interfere with your everyday life?	2.91 (1.98)
ICIQ score	3.0 (3.8)

^1^n (%); Mean (SD)

**Fig. 1:**
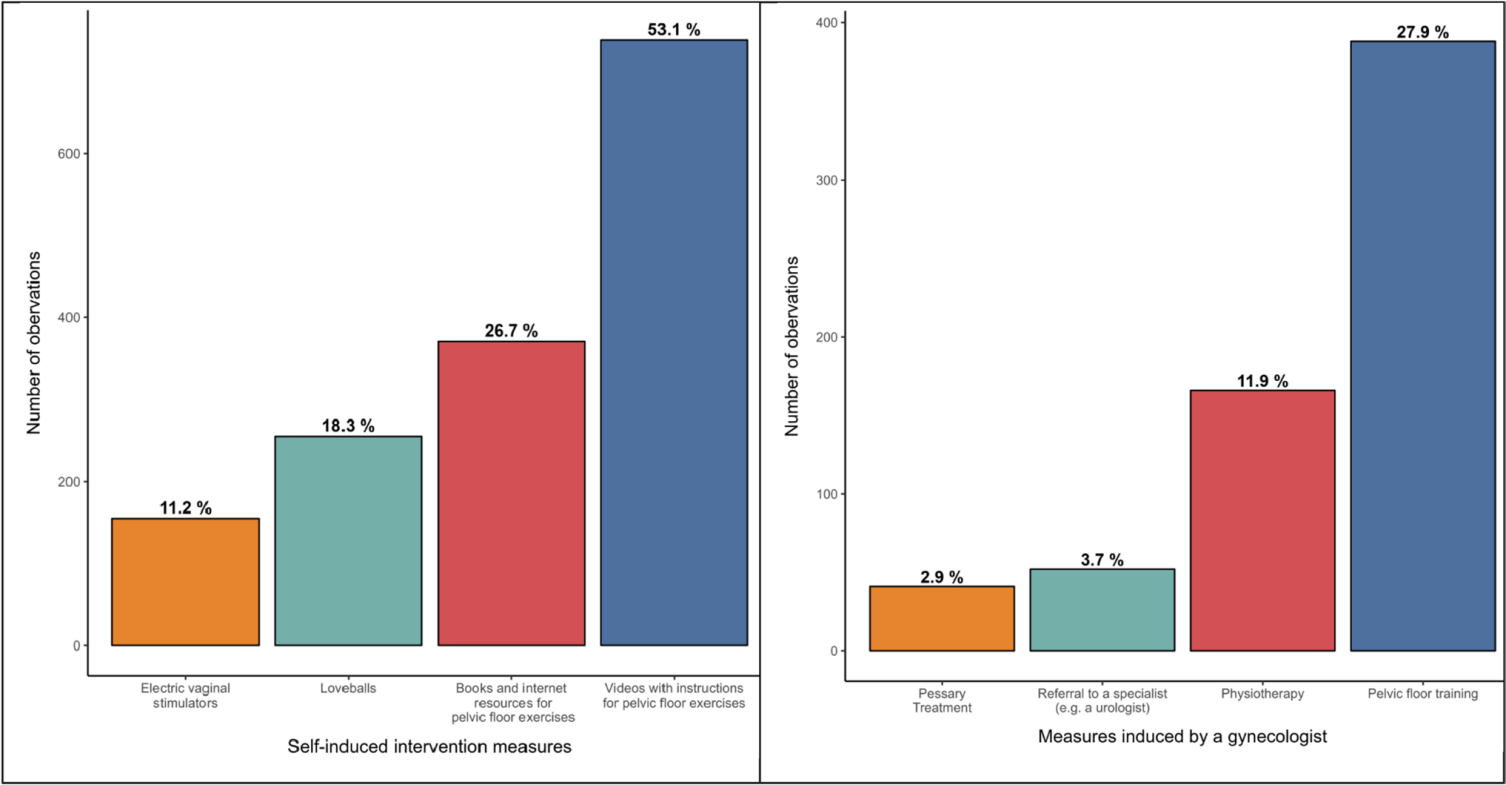
Self-induced intervention measures versus measures induced by a gynecologist

We asked the participants of the survey which kind of self-induced measures they have performed. Figure 1 illustrates self-induced measures as well as measures induced by the participants’ gynecologists in all women, who reported any incontinence symptoms. Overall, 79.3 (%) of the participants of the survey with UI underwent measures to deal with the complaints. In total, 155 of 1390 (11.2%) used electric vaginal stimulators. Interestingly, 371 out of 1020 participants (26.7%) used books and internet resources to get instructions for pelvic floor exercises. While 18,3% of the participants (*n* = 255) used loveballs, more than a half (53.1%, *n* = 738) used videos with instructions for pelvic floor exercises.

We further analyzed whether the need for self-induced intervention is influenced by other factors, such as by the level of education, age or ICIQ_SF Score. We were able to show that ICIQ-SF Score was significantly associated with all self-induced measures. High School diplomas and academic degrees were associated with the use of love balls (*p* < 0.05). If, in addition to incontinence, the impairment of sexuality due to pelvic floor disorders (PISQ-IR Condition Impact) is included in the multivariate model, it becomes apparent that the influence of the ICIQ-SF Score is no longer significant, whereas the PISQ-IR is significant in sexually active women (Table 3).

**Table 3:** Implementation of measures in relation to urinary incontinence and sexual functioning

	Self-therapy without assistive devices									Self-therapy with assistive devices								
	Only ICIQ-SF *n* = 1236			Not sexually active *n* = 278			Sexually active *n* = 732			Only ICIQ-SF *n* = 1236			Not sexually active *n* = 278			Sexually active *n* = 732		
Characteristic	OR^1^	95% CI^1^	p-value	OR^1^	95% CI^1^	p-value	OR^1^	95% CI^1^	p-value	OR^1^	95% CI^1^	p-value	OR^1^	95% CI^1^	p-value	OR^1^	95% CI^1^	p-value
ICIQ-UI SF Score	1.10	1.05, 1.14	< 0.001	1.07	0.97, 1.18	0.2	1.06	1.00, 1.13	0.047	1.10	1.06, 1.15	< 0.001	1.09	0.99, 1.20	0.081	1.05	0.99, 1.11	0.10
Current age	0.99	0.96, 1.02	0.4	1.02	0.96, 1.08	0.6	0.99	0.95, 1.02	0.4	1.02	0.99, 1.05	0.2	1.06	0.98, 1.13	0.13	1.01	0.97, 1.05	0.5
Level of Education																		
Intermediate School-Leaving Certification	1.99	0.68, 5.89	0.2	5.22	0.85, 36.5	0.078	2.49	0.52, 12.0	0.2	0.30	0.10, 0.89	0.030	0.17	0.02, 1.10	0.066	0.31	0.06, 1.45	0.14
General/Subject-Related Qualification for University Entrance	1.66	0.59, 4.70	0.3	3.30	0.62, 19.7	0.2	1.68	0.38, 7.52	0.5	0.28	0.10, 0.79	0.016	0.28	0.05, 1.52	0.14	0.24	0.05, 1.07	0.065
University/University of Applied Sciences Degree	2.15	0.77, 6.02	0.14	2.85	0.56, 16.4	0.2	2.48	0.56, 11.0	0.2	0.30	0.10, 0.83	0.020	0.25	0.04, 1.32	0.10	0.25	0.05, 1.09	0.069
PISQ-IR: Not Sexually Active—Condition Impact				1.35	0.97, 1.90	0.078							1.27	0.91, 1.77	0.2			
PISQ-IR: Sexually Active—Condition Impact							0.72	0.55, 0.94	0.018							0.65	0.51, 0.83	< 0.001
R^2^ (*Nagelkerke*)	0.02			0.04			0.02			0.02			0.05			0.03		

^1^OR = Odds Ratio, CI = Confidence Interval

## Discussion

We report the results of an anonymous social media-based survey assessing PFD and the needs of women up to 5 years postpartum. We were able to show that 48.4% of the women had urinary incontinence with varying frequency. Former published data estimated an even lower prevalence of postpartum urinary incontinence in women with a wide range between 3 and 40% [8]. Fecal incontinence seems to be very rare in our study population with 5.7%. According to published data, 2.4% to 8.0% of postpartum women suffer from involuntary loss of formed stool [9–11]. Differences can be explained by differences in study design, subgroups and populations being studied.

Interestingly, measures induced by the outpatient gynecologist were pelvic floor training, physical therapy, referral to a urologist and pessary therapy. Pelvic floor muscle training is known to be effective in the treatment of pelvic organ prolapse in comparison to no treatment [6]. Current studies also show that postpartum pelvic floor muscle training decreases the rate of urinary incontinence 6 months after confinement [2], but the long-term efficacy of postpartum pelvic floor muscle training is not clear [12].

Burkhart et al. reported that about 2/3 of their participants have no awareness of pelvic floor rehabilitation to address PFD [13]. However, a part of the population seeks further solutions for their complaints. A relevant number of participants in this study used different sources (books, internet, videos) to receive information about pelvic floor exercises, which stresses the necessity for information about the treatment or prevention of PFD [14]. The use of loveballs shows that a part of the study population seems to be open minded to alternative, non-conventional methods.

Our study shows that the majority of patients with PFD has not been asked about having (typical) PFD complaints and a relevant amount did not feel taken seriously by their gynecologist. These results highlight the need for new guidelines or policies that address the questioning of symptoms related to PFD after delivery. Another mandatory examination at 3 months post-partum seems conceivable, analogous to the maternity records of the Federal Joint Committee (G-BA). Both UI and PFD could be explicitly addressed at that time. The large number of patients undergoing self-induced measures shows the unmet intervention need.

Interestingly, ICIQ-SF Score was associated with the use of all queried self-induced measures, which shows the relation between patient-reported complaints and the intervention. The use of love balls was associated with a higher educational level.

A previously published study shows that the level of education and willingness to participate in a postpartum pelvic floor muscle training program is significantly related, which is in line with our results [15]. In the context of interpretating results, it has to be mentioned that approximately 89,5% of our study members have at least general or subject-related qualifications for university entrance with 62,6% holding a form of university degree, which is reflecting a well-educated study population. The adaption of the model by considering sexuality shows that this restriction of sexuality may even be more burdensome for the women than incontinence alone, as it is then associated to a higher degree with the utilization of measures.

Even though a large number of respondents could be recruited through social media, a possible selection bias cannot be completely excluded as described before. However, the fact that the data are approximately normally distributed in many variables speaks for a broad coverage of the target population. The low number of missing values shows the willingness to provide information on these topics as well. The low variance explanation by the multivariate models shows that there are further influencing factors beyond the variables considered. However, due to the complexity of predicting behavior, this was to be expected.

## Conclusion

The presented study exhibits the unmet needs of postpartum women and their willingness to undergo self-induced measures. Consequently, postpartum PFD should be addressed more frequently in the outpatient setting. Additionally, more systematic information about the treatment of postpartum PFD could help to address unmet care needs and improve interventions.
